# Origin and fate of A/H1N1 influenza in Scotland during 2009

**DOI:** 10.1099/vir.0.039370-0

**Published:** 2012-06

**Authors:** Samantha Lycett, Nigel J. McLeish, Christopher Robertson, William Carman, Gregory Baillie, James McMenamin, Andrew Rambaut, Peter Simmonds, Mark Woolhouse, Andrew J. Leigh Brown

**Affiliations:** 1 Institute of Evolutionary Biology, University of Edinburgh, Ashworth Laboratories, Edinburgh EH9 3JT, UK; 2 Centre for Immunity, Infection and Evolution, Ashworth Laboratories, West Mains Rd, Edinburgh EH9 3JT, UK; 3 Department of Mathematics and Statistics, University of Strathclyde, 26 Richmond Street, Glasgow G1 1XH, UK; 4 Health Protection Scotland (HPS), 3 Clifton Place, Glasgow G3 7LN, UK; 5 West of Scotland Specialist Virology Centre, Gartnavel General Hospital, Glasgow, UK; 6 Wellcome Trust Sanger Institute, Wellcome Trust Genome Campus, Hinxton, Cambridge, CB10 1SA, UK

## Abstract

The spread of influenza has usually been described by a ‘density’ model, where the largest centres of population drive the epidemic within a country. An alternative model emphasizing the role of air travel has recently been developed. We have examined the relative importance of the two in the context of the 2009 H1N1 influenza epidemic in Scotland. We obtained genome sequences of 70 strains representative of the geographical and temporal distribution of H1N1 influenza during the summer and winter phases of the pandemic in 2009. We analysed these strains, together with another 128 from the rest of the UK and 292 globally distributed strains, using maximum-likelihood phylogenetic and Bayesian phylogeographical methods. This revealed strikingly different epidemic patterns within Scotland in the early and late parts of 2009. The summer epidemic in Scotland was characterized by multiple independent introductions from both international and other UK sources, followed by major local expansion of a single clade that probably originated in Birmingham. The winter phase, in contrast, was more diverse genetically, with several clades of similar size in different locations, some of which had no particularly close phylogenetic affinity to strains sampled from either Scotland or England. Overall there was evidence to support both models, with significant links demonstrated between North American sequences and those from England, and between England and East Asia, indicating that major air-travel routes played an important role in the pattern of spread of the pandemic, both within the UK and globally.

## Introduction

The determinants of influenza transmission and spread, particularly in the pandemic phase, are of critical importance for strategies to mitigate disease outcome (Ferguson *et al.*, 2006). A dominant feature of current thinking is the gravity model, whereby the largest population centres determine the pattern of spread. This has been shown to apply to both the 1918 pandemic and recent seasonal influenza (Eggo *et al.*, 2011; Viboud *et al.*, 2006a). Indeed, contemporary reports suggested that even the 1890 pandemic followed a pattern within Great Britain compatible with this model (Parsons, 1891; Robertson & Elkins, 1890). However, an additional model that emphasizes the role of air travel in transmission and spread of influenza has been proposed (Brownstein *et al.*, 2006). Scotland has a total population of 5.2 million from a UK total of 62 million, and its two largest conurbations, Greater Glasgow (1.2 million) and Edinburgh (0.48 million), comprise between them just 10 % of the population of London and the South East of England (16.5 million) (Office for National Statistics, 2010). The gravity model thus predicts that the population concentration in the London metropolis will dominate the rest of the UK. However, air travel from provincial centres, and particularly from Scotland, is increasingly routed independently of London. The 2009 H1N1 pandemic, which led to 69 deaths in Scotland and 387 in England and Wales up to April 2010 (Health Protection Agency, 2010), provides an unparalleled opportunity to examine these competing hypotheses of influenza transmission in the UK, information of considerable value for future pandemic planning (Viboud *et al.*, 2006b).

The first virologically confirmed case of A H1N1 influenza in Scotland with a clear travel link to Mexico was reported on 27 April 2009. Virological confirmation of all cases continued until the end of the ‘containment phase’ (2 July 2009), by which time 1409 cases of A H1N1 2009 had been confirmed in Scotland, along with 10 cases of influenza A H3N2. GP consultation rates for influenza-like illnesses were, however, relatively constant over the period to the end of July 2009, but did not show the usual summer decline (Health Protection Scotland, 2011). The school-holiday period is taken as the end of the summer phase of the pandemic (Health Protection Agency, 2010). Cases began to increase again in August and September, more rapidly in eastern Scotland, and the winter phase in Scotland peaked in late November 2009 (Health Protection Scotland, 2011; McLeish *et al.*, 2011).

In order to assess the origin and outcome of introductions of pandemic H1N1 into Scotland during the two phases of the pandemic in 2009, we obtained viral genome sequences of 70 strains distributed equally between the summer and winter phases, and sampled representatively (as far as possible) with respect to geographical location. Phylogenetic analysis of these strains in comparison with strains from England and from other global sources reveals different epidemic patterns in the summer and winter phases, with no particularly close links to English sequences in the winter phase, in contrast to what was seen in the summer phase of the pandemic. The potential for distinction of Scottish influenza epidemics from concurrent infection in England should be noted for future pandemic planning purposes.

## Results

Influenza virus genome sequences for a total of 70 high-quality genomes from Scottish samples were obtained. Table 1 indicates their distribution by Health Board and pandemic phase (summer or winter), aggregating dates and locations as necessary to maintain confidentiality. These sequences were aligned with a sample of 293 genomes downloaded (2 February 2011) from the NCBI Influenza Virus Resource (Bao *et al.*, 2008). We randomly chose one sequence per country per month, except for the USA, China and Russia, where one sequence was chosen per place (city or region) per month. We additionally included 136 recently sequenced virus genomes from England, four from Scotland and seven from Ireland from the study by Baillie *et al.* (2012) to give a total of 510 strains. Identical sequences collected on the same day were then removed from further analyses, resulting in a final dataset of 492 sequences (Table 2).

**Table 1.** Pandemic phase and location (by Health Board) for 70 sequenced pandemic H1N1 influenza strains from Scotland

Health Board	Pandemic phase	
	Summer*	Winter†
Ayrshire and Arran	2	0
Borders	1	0
Tayside	0	18
Lothian	3	3
Fife	3	0
Forth Valley	1	0
Greater Glasgow	14	4
Grampian	0	14
Highland	1	0
Lanarkshire	6	0
**Total**	**31**	**39**

* Two in May, 28 in June, one in July 2009.

† Nine in October, 23 in November, six in December 2009, one in February 2010.

**Table 2.** Sequence origin for phylogenetic analysis

Location	Summer	Winter	Total
Scotland (all)	33	39	72
RUK	102	26	128
Europe	13	33	46
North America	80	44	124
South America	14	7	21
Asia	42	49	91
Oceania	4	3	7
Africa	0	3	3
**Total**	**288**	**204**	**492**

**Fig. 1.**
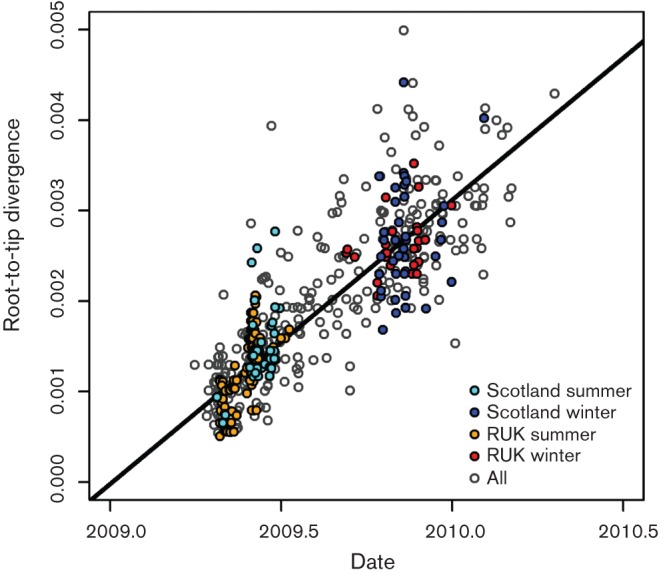
Evolutionary divergence during the influenza A 2009 H1N1 pandemic. Root-to-tip plot of evolutionary divergence based on the ML tree in Fig. S1, plotted against elapsed time.

**Fig. 2.**
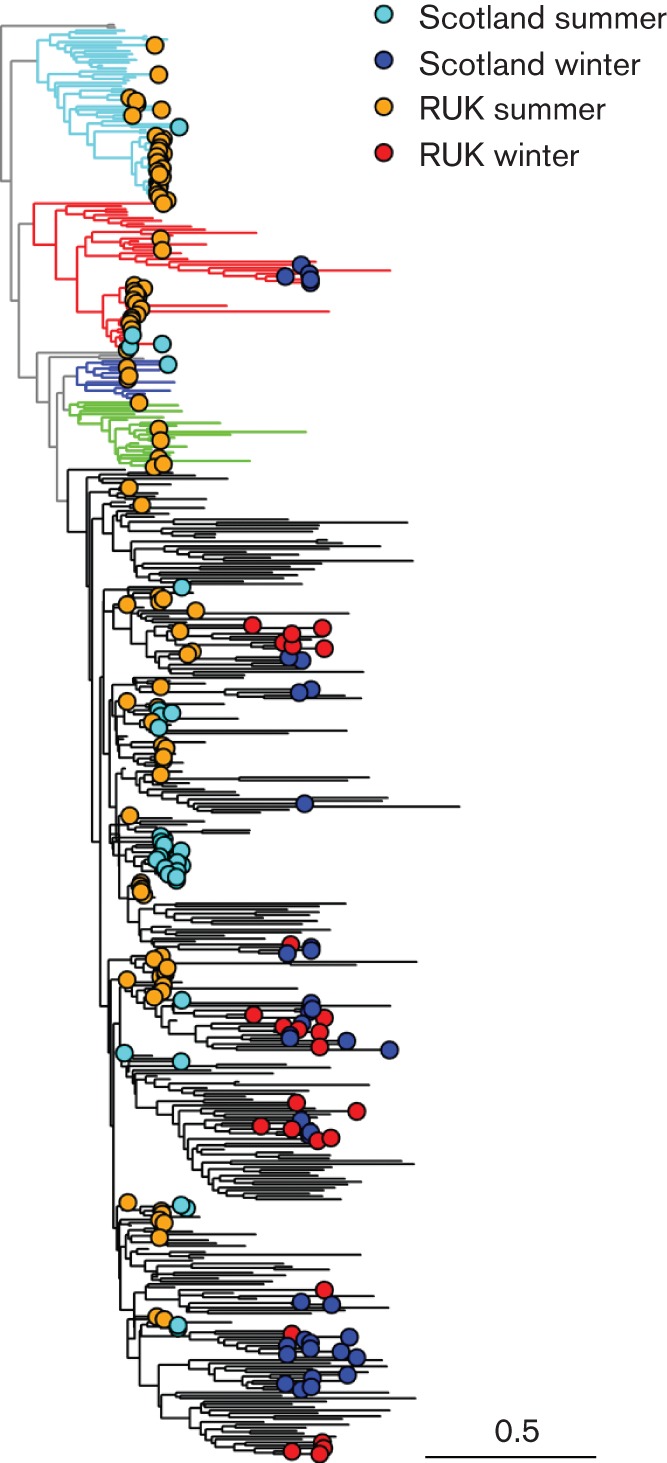
Time-resolved phylogenetic tree of influenza A H1N1 2009 sequences. Time-resolved phylogeny produced in beast for seven-segment sequences from Scotland, RUK and global sequences listed in Table 2. See Fig. S3 for strain labels. Global sequences are not shown for clarity. Branch colours: cyan, global clade 2 (Nelson *et al.*, 2011b); red, global clade 3; blue, global clade 5; green, global clade 6; black, global clade 7. Bar, 0.5 years.

The global sequence diversity exhibited by pandemic H1N1/2009 has been divided into seven major clades (Nelson *et al.*, 2011b). Initial phylogenetic analysis by maximum likelihood (ML) revealed that influenza virus sequences from Scotland were distributed across the global tree, indicating a series of introductions from which the virus spread locally, rather than a single point source introduction in each phase (Fig. S1, available in JGV Online). The overall total evolutionary change was restricted as revealed by a root-to-tip plot (Fig. 1), but even with a divergence of as little as 0.3 % occurring over the 1 year observation period, there was a very strong molecular-clock effect. Comparison of early and late sequences for three regional categories, Scotland, rest of UK (RUK) and North America, showed clear and significant divergence between sequences from the summer and winter phases of the epidemic (Fig. S2). Despite the small amount of evolution that occurred even globally within the summer phase, there was clear clustering of Scottish sequences into a series of clades that were not split by global sequences. As all samples analysed have accurate date information, the 492 strain dataset was analysed using time-resolved phylogenetic methodology implemented in the beast package (Drummond & Rambaut, 2007) to enhance resolution (Figs 2 and S3). The results were highly concordant with the ML tree overall, with minor differences such as might be expected given the low level of support for many individual clades.

### Summer phase

The most striking feature of the summer epidemic in Scotland was that almost half (15/33) of the sequences fell into a single clade in both the ML (Fig. S1) and the 50 % consensus beast (Figs 2 and S3) trees, suggesting a point introduction followed by rapid local spread. The samples represented in this clade are predominantly from Glasgow postcodes, but also extended to include postcodes PA23 (Dunoon) in the Highland Health Board and PA4, central Paisley, and were obtained between 30 May and 26 June 2009. The cluster included two cases for which a travel link to North America existed (450 and 483), but the location of each of these strains within the cluster strongly supports an indigenous rather than a travel-associated source for their infections. The clade has an apparent link in both the beast and ML trees, with some infections identified in London as early as 13 May and Birmingham from 1 May, suggesting an introduction from England. The ML tree included another pair of sequences in this clade (477, 487) that came from a clustered outbreak in Lanarkshire, whilst the beast tree located them near a separate group of sequences from England. The low support for any particular grouping renders the alternatives difficult to distinguish, but the introduction in either case seems to be from England. A group of three Scottish strains forming another clade (442, Largs; 459, Glasgow; and 481, Clackmannan) are similarly phylogenetically associated with a sample from London in both trees, and another pair, 457 and 462, which were tentatively indicated as travel cases owing to links with the USA and Mexico, respectively, are linked closely to a substantial clade centred in London in May 2009, revealing the extra information provided to surveillance studies from sequence characterization. This cluster belongs to global clade 3; all of the others belong to global clade 7 (Nelson *et al.*, 2011b). Finally, of the five sequences with no link to any other in our dataset, two (490, 494) had clear links with English sequences and three had direct links with North America but possible links to England as well; again, these form part of global clade 7.

### Winter phase

The phylogenetic pattern of Scottish influenza virus sequences in the winter phase of the pandemic was rather different (Figs 2 and S3). Instead of domination by a single clade, several clades of similar size were circulating within the same infected community. Two of these clades appeared to originate in North America (based around strains 514 and 509, respectively), and another probably did (based around 529). Together these accounted for 18/39 (46 %) of the sequenced strains. A more diverse group (including 507) and several small groups or clades that made up the remainder of the sequences had direct links with sequences from England. The clade that includes strain 514 belongs to global clade 3, and all others to clade 7, as before (Nelson *et al.*, 2011b).

### Phylogeography

**Fig. 3.**
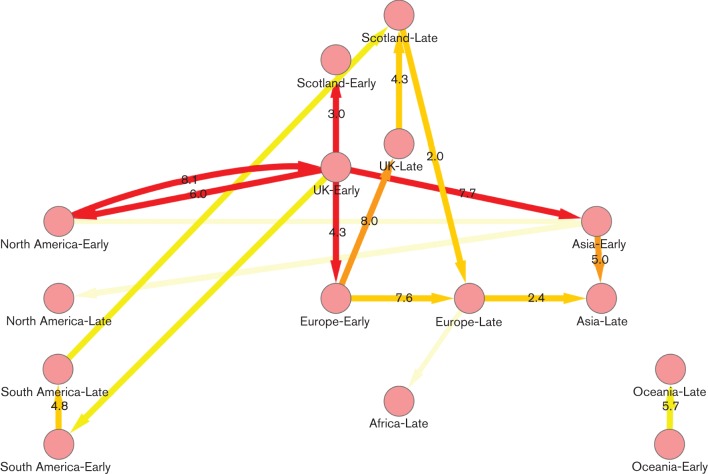
Migration rates of influenza A H1N1 2009 strains between Scotland, RUK and continents. Rates were estimated using beast trees and a discrete traits model. The diagram was created by combining five independent BSSVS discrete trait runs of 10 000 000 steps each, sampling every 1000 with a 10 % burn in. Edge values represent the median transition rate year^−1^ (calculated as the median of the individual rate×the overall discrete clock rate). Only rates with indicator probability ≥0.5 are shown. Edge colours represent the mean of the indicator probabilities across the runs: palest yellow, 0.5–0.6; yellow, 0.6–0.7; pale orange, 0.7–0.8; deep orange, 0.8–0.9; red, 0.9–1.0.

The pattern of strain exchange between Scotland, RUK and the major continents was analysed using a discrete trait-mapping approach (Bayesian stochastic search variable selection; BSSVS) in beast (Lemey *et al.*, 2009, 2010). This approach quantifies the geographical patterns visible in the phylogeny described above and indicates which associations can be considered robust. The numbers of sequences from each population and phase (summer/winter) analysed are shown in Table 2. The diagram was created by combining five independent BSSVS discrete trait runs (see Methods). The network map (Fig. 3; highest posterior density plots given in Fig. S4) confirms the strong and significant links between UK-Early and Scotland-Early sequences, revealing, as indicated, that many (although not all) of the initial introductions were from England. Whilst travel-associated cases were described in Scotland, these did not lead to significant onward transmission. The link between these two for the winter phase has a higher median rate of 4.3 vs 3.0, but a lower probability (0.7–0.8 vs 0.9–1.0). Very strong links are supported between the North American and UK-Early sequences, which are bidirectional, with a unidirectional link from the latter to Asia-Early (rate 7.7, probability 0.9–1.0), a noticeably stronger link than that from North America. Interestingly, there is no significant link between UK-Early and UK-late, any more than there is between Scotland-Early and Scotland-Late. Rather, the UK summer strains fed the Europe-Early strains (rate 4.3, probability 0.9–1.0), which then connected to the UK winter epidemic (rate 8.0, probability 0.8–0.9), whilst the Scotland winter population appears to feed into Europe (rate 2.0, probability 0.8–0.9). Overall, a very high degree of interconnectedness between global centres is revealed by this analysis.

**Fig. 4.**
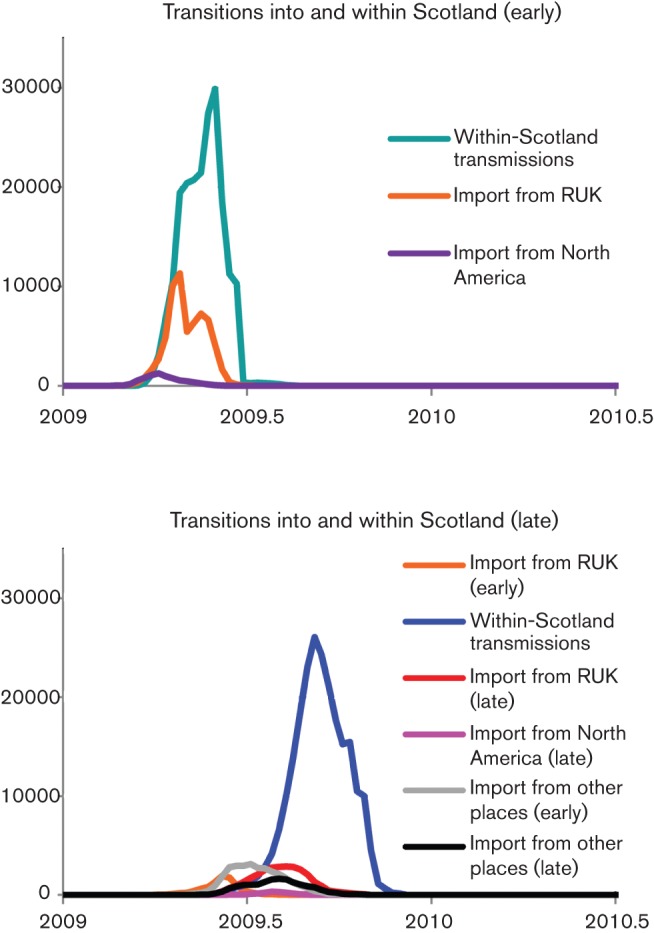
Distribution of transmission times into and within Scotland. Distribution of earliest transmission event times in weekly bins (1/52 of a year) for events within Scotland, and between other locations and Scotland, as inferred from a subsample of 5000 beast output trees with a discrete traits model from five independent runs. (a) Distribution of events into and between Scotland for the summer phase (Scotland-Early); (b) distribution of events into and between Scotland for the winter phase (Scotland-Late). The *y*-axis represents the number of transmission events aggregated for the all of the trees in the sample; distribution line colours indicate the origin of the events, and only those with visible traces are plotted. Light blue, Scotland summer; dark blue, Scotland winter; orange, RUK summer; red, RUK winter; dark purple, North America summer; magenta/pink, North America winter; grey, other summer; black, other winter.

The analysis given above quantifies the importance of specific geographical origins, but does not extend to the subsequent epidemics developing from these introductions. However, given that each sample came from a different individual, it is possible to use the beast output to quantify the number and time distribution of transmissions within countries (Lewis *et al.*, 2008). We have therefore performed such an analysis to determine the relative importance of strain import to Scotland from RUK compared with local epidemic spread, for the summer and winter phases (Fig. 4a, b, respectively; an equivalent plot for RUK is shown in Fig. S5). In the summer phase, the introduction of viruses from North America can be seen as an early event that was quickly replaced by import from the RUK, which remained a substantial but declining source of transmissions in Scotland during this phase. In winter, however, there is some evidence for summer–winter transmission within the UK, but import from RUK is not more important than from outside, and both are rapidly dominated by what became a genetically clearly differentiated epidemic within Scotland.

## Discussion

Through recent technological and bioinformatic advances in virus genetic analysis and internationally coordinated surveillance, the 2009 influenza A pandemic has presented a unique opportunity to analyse the global spread of a new virus strain and to explore different transmission models, such as local spread and introductions from outside, in the dissemination of infections in a community. We have previously shown using highly specific diagnostic assays that the virus had infected approximately 20 % of the adult population of Scotland by 1 December 2010 (McLeish *et al.*, 2011). From the same study, it is possible to estimate a clinical attack rate of approximately 10 % for this virus, rendering classical epidemiological contact tracing almost impossible. Here we have added to the epidemiological information a phylogenetic analysis based on newly generated sequences of seven out of eight segments of the viral genome from a total of 70 strains from Scotland. These strains were distributed equally between the summer and winter phases and sampled representatively with respect to geographical location. Comparison of these strains with 128 strains from RUK and 292 from other global sources revealed strikingly different epidemic patterns within Scotland in the summer and winter phases. The initial phase was characterized by multiple independent introductions from both international and other UK sources, with significant local expansion of one clade that had a clear link to a possible progenitor in England, whilst the winter phase was more diverse genetically with several clades of similar size, some of which had no particularly close phylogenetic affinity to strains circulating in England. Bayesian phylogeographical analysis confirmed that the links between the epidemic in other parts of the UK to Scotland were stronger in the summer than in the winter phase.

Early studies of the diversification of A H1N1 2009 revealed seven distinguishable clades of virus (Nelson *et al.*, 2009), six of which were identified in North America in the summer phase (Nelson *et al.*, 2011b). As expected, the samples from Scotland included only a subset of the global diversity, with just two clades, 3 and 7, being represented. However, whilst clade 3 virtually disappeared from the USA by winter (Nelson *et al.*, 2011b), it still comprised 13 % (5/39) of the winter-phase sample in Scotland.

In comparison to England, Scotland had much lower diversity in the summer phase, with a smaller number of introductions and extensive local spread in the greater Glasgow area, leading to an epidemic dominated by one large clade. However, this clade did not lead into the winter epidemic, which appeared to arise from separate introductions. To put this on a quantitative basis, we used the beast output files to evaluate the extent to which the epidemic in Scotland developed independently (Fig. 4). This demonstrated clearly that local spread was dominant in both phases, but that the winter phase (Fig. 4b) appeared to be completely distinct from the epidemic in England. Unfortunately, the representation of the two phases among strains from England was quite uneven, with only 26 (out of 147) strains sequenced coming from the winter phase. This low sampling density means that we cannot say more. Nevertheless, it appears that the gravity model alone does not fully explain the phylogenetic pattern in the two phases of the epidemic.

A surprising feature of the results that may be relevant, however, is the clear links between the UK-Early sequences and both North America and Asia, particularly in the summer phase, strongly suggesting an epidemiological link from North America to Asia through the UK in the global spread of the pandemic. This suggests that air-travel routes influenced the initial spread of the pandemic between continents, as there was a much weaker direct connection between the sequences from these continents in contrast to the results obtained by Jombart *et al.* (2009) from haemagglutinin and neuraminidase sequences obtained in the summer phase. In a phylogeographical analysis of the summer and winter phases of the 2009 pandemic in the USA, Nelson *et al.* (2011b) found evidence for founder effects leading to phylogenetically distinct epidemics among different cities in the summer phase, whereas the winter phase was almost completely homogeneous across the USA. The high degree of mixing occurring within the USA was illustrated very clearly by the analysis of a single university campus, implying >20 phylogenetically distinct introductions to that one site in the winter phase (Holmes *et al.*, 2011). Earlier studies of seasonal influenza suggested seeding from the tropics as an important influence in the establishment of annual epidemics (Rambaut *et al.*, 2008), but the phylogeographical analysis here does not suggest that any particular link outside the UK was important for the winter epidemic in Scotland. Further and more detailed sample collections are clearly required to provide adequate answers to the questions of persistence through summer and the source of winter epidemics within the UK.

## Methods

### 

#### Strain selection.

Approximately equal numbers of samples were selected for sequencing from the summer (May–July) and winter (September–February) phases of the 2009 pandemic. For the containment phase (to 2 July 2009), samples had been stored in the Specialist Virology Centre and WHO National Influenza Centre, Gartnavel General Hospital, for all confirmed cases. Samples were selected for sequencing to provide representation of all travel-associated cases and all geographical clusters identified by the HPS during the containment (summer) phase, which was concentrated in the west of Scotland (Health Protection Agency, 2010). The same level of detailed knowledge was not available for the winter phase, as only hospitalized cases and GP sentinel cases were reported to HPS after the end of the containment phase. Based on that information (Adamson *et al.*, 2010), there was a difference between the summer phase and the winter phase, with proportionately more hospitalized cases in Health Boards from eastern Scotland in the winter phase than were recorded in the summer phase (Fig. S6). Winter-phase samples were obtained primarily from the Specialist Virology Laboratory in Edinburgh and the WHO National Influenza Centre, Department of Medical Microbiology, in Aberdeen.

#### Sample processing.

All sequences were obtained by direct amplification from clinical swabs. Samples were selected for which the original quantitative PCR *C_t_* value was ≤30 to increase the probability of successful amplification.

##### Amplification.

RNA from clinical samples was reverse-transcribed and amplified using the all-segment PCR method of Zhou *et al.* (2009). Forward and reverse primers common_uni12 (5′-GCCGGAGCTCTGCAGATATCAGCRAAAGCAGG-3′) and common_uni13 (5′-GCCGGAGCTCTGCAGATATCAGTAGAAACAAGG-3′), respectively, were used in a single reaction to amplify all eight segments of the influenza A genome using published protocols (Baillie *et al.*, 2012). Amplification reactions were performed in 100 µl volumes containing 1× SuperScript III One-Step RT-PCR buffer (Invitrogen), 0.5 µM forward and reverse primers, 2 µl SuperScript III reverse transcriptase/Platinum *Taq* High Fidelity enzyme mixture (Invitrogen) and 10 µl RNA extracted from clinical material. Thermal-cycling parameters were as described previously (Baillie *et al.*, 2012). The cDNA generated by RT-PCR was amplified further in 86 reactions in 96-well plates by a primer-walking method using Phusion Hot Start High-Fidelity DNA polymerase (Finnzymes/NEB) and previously described primers (Baillie *et al.*, 2012). Reactions were performed in 20 µl volumes with a mineral oil overlay and contained 1 µl cDNA, 200 µM each dNTP, 0.5 µM forward and reverse primers and 0.02 U Phusion Hot Start High-Fidelity DNA polymerase µl^−1^, using the thermal-cycling conditions described by Baillie *et al.* (2012). PCR products were sequenced directly using Sanger sequencing chemistry on an ABI capillary sequencer (Baillie *et al.*, 2012).

##### Assembly.

Sequence fragments were assembled into ‘contigs’ using Phrap (http://www.phrap.org/). The initial contigs were trimmed automatically by quality score using the trace2seq.pl script (which uses Phred, http://www.phrap.org/) to generate the contig files from the raw sequence chromatogram files. These ‘contigs’ were roughly aligned to a reference complete genome (A/England/195/2009) using a custom java script, before detailed alignment using the Simmonics software v. 1.9 (Simmonds & Evans, 2011) and final manual editing.

A total of 102 genome sequences were obtained in this way. The sequences were also assembled using a custom assembler designed for short sequence reads based on word matching (‘Kmer15’) in an attempt to increase sequence length in low coverage regions, particularly PB1 (mean coverage 21 %). However, after the assembly process, the coverage (and quality) on PB1 (segment 2) was still poor, so only segments 1 and 3–8 were used. We also only included samples that had ≥90 % coverage on each of segments 1 and 3–8. This resulted in the use of 70 high-quality seven-segment sequences, with mean coverage (per segment) of >98 %. The sequences have been submitted to GenBank under accession numbers CY107183–CY107823.

##### Phylogenetic analysis.

Bootstrapped ML trees were obtained using using RAxML (Stamatakis, 2006) with the GTR+Γ substitution model (general time-reversible plus discretized gamma distribution of rates over sites) and 1000 bootstraps.

Time-resolved phylogenetic trees were obtained using beast 1.6.1 (Drummond & Rambaut, 2007) with the GTR+Γ substitution model, strict molecular clock and either constant population size or exponentially growing effective population size. Due to the lack of genetic diversity, rapid initial spread of the virus, global sampling and large number of sequences, more parameter-rich models, such as the relaxed molecular clock or skyride tree prior, were not appropriate.

A final sample, consisting of 8804 trees inferred with a strict clock and exponential population size, was summarized as a maximum clade credibility tree and used in further analysis. This sample was the product of four independent runs, each of 50 000 000 (runs 1 and 2) or 100 000 000 (runs 3 and 4) samples and using a burn in of 20 000 000 samples per run, resulting in a posterior effective sample size (ESS) of >400 and a likelihood ESS of >1000. This set of trees was further subsampled by a factor of 10 to 881 trees (posterior ESS >200) for use in the subsequent phylogeographical analysis.

#### Phylogeographical analysis.

The rates of transition from one region (e.g. country or continent) to another were calculated within beast, by inferring models of discrete trait evolution upon the sample of trees using an asymmetrical model (Lemey *et al.*, 2009; Nelson *et al.*, 2011a). Additionally, BSSVS was used in order to determine which rates were significant (Lemey *et al.*, 2009). An asymmetrical discrete traits model was fitted upon the set of 881 trees using 10 000 000 Markov chain Monte Carlo (MCMC) steps and sampling every 1000 steps. The resulting transition rates were summarized with the Rate Indicator tool (part of the beast package), and rates that were used in the models for at least 50 % of the MCMC steps (equivalent Bayes factor >10) were considered significant. Five independent BSSVS discrete trait runs of 10 000 000 steps each, sampling every 1000 with a 10 % burn in, were combined.

We extracted the times of transmission from other regions into Scotland, and within Scotland itself, from the set of beast output trees; these are annotated with the inferred ancestral states (country or continent) at each internal node as part of the discrete traits model. We used a subsample of 5000 trees (1000 per run, taken post-burn in) from the five independent discrete trait runs, and recorded the time and inferred geographical origin of the immediate ancestors to every node (internal or tip) with a Scottish state. These node times represent the earliest transmission dates from the ancestral state into a Scottish state (the latest time would be the time of the Scottish child node). The number of transmissions into and within Scotland, for the summer and winter phases (i.e. Scotland-Early and Scotland-Late states), were collated into weekly bins (1/52 of a year) in order to produce the distributions of regional transmission events.
